# Peptide-Drug Conjugates and Their Targets in Advanced Cancer Therapies

**DOI:** 10.3389/fchem.2020.00571

**Published:** 2020-07-07

**Authors:** Paul Hoppenz, Sylvia Els-Heindl, Annette G. Beck-Sickinger

**Affiliations:** Faculty of Life Sciences, Institute of Biochemistry, Leipzig University, Leipzig, Germany

**Keywords:** peptide, GPCR, cancer, peptide-drug conjugate, boron neutron capture therapy

## Abstract

Cancer became recently the leading cause of death in industrialized countries. Even though standard treatments achieve significant effects in growth inhibition and tumor elimination, they cause severe side effects as most of the applied drugs exhibit only minor selectivity for the malignant tissue. Hence, specific addressing of tumor cells without affecting healthy tissue is currently a major desire in cancer therapy. Cell surface receptors, which bind peptides are frequently overexpressed on cancer cells and can therefore be considered as promising targets for selective tumor therapy. In this review, the benefits of peptides as tumor homing agents are presented and an overview of the most commonly addressed peptide receptors is given. A special focus was set on the bombesin receptor family and the neuropeptide Y receptor family. In the second part, the specific requirements of peptide-drug conjugates (PDC) and intelligent linker structures as an essential component of PDC are outlined. Furthermore, different drug cargos are presented including classical and recent toxic agents as well as radionuclides for diagnostic and therapeutic approaches. In the last part, boron neutron capture therapy as advanced targeted cancer therapy is introduced and past and recent developments are reviewed.

## Need for Targeted Cancer Therapy

For a long time, cardiovascular diseases were the leading cause of death among middle-aged adults globally. This changed recently in high-income countries, where cancer is now responsible for twice as many deaths as cardiovascular diseases (Dagenais et al., 2019). In 2018, cancer was responsible for 9.6 million deaths and 18.1 million new cases were diagnosed globally (Bray et al., 2018). The most frequently occurring cancer in women is breast cancer and in men, prostate cancer is most common. Notably, the leading cause of death is lung cancer in both sexes (Siegel et al., 2019). It is predicted that these numbers will rise in the future and 27.5 million new cases of cancer will be diagnosed worldwide each year by 2040 (Bray et al., 2018). This trend clearly demonstrates that novel therapeutic approaches have to be developed and efforts have to be strengthened in the future to improve the efficacy of already existing treatments.

For the last 60 years, chemotherapy remained the trademark of cancer treatment (Gilman, 1963; DeVita and Chu, 2008). In this approach, highly cytotoxic drugs are systemically administered, which seek into rapidly dividing cancer cells. However, these chemotherapeutic drugs affect also healthy cells that exhibit high proliferation rates like intestinal epithelium cells. This peripheral toxicity is the reason for frequently occurring side effects, which can vary from hair loss, anemia, bruising and bleeding to neuropathy and many more (Strebhardt and Ullrich, 2008). The occurrence of undesired effects depends highly on the patient and the applied chemotherapeutic drug. This holds not only true for chemotherapeutics, but rather for any therapeutically active molecule that is administered systemically without bearing any selectivity providing features.

One possibility to reduce side effects is to waive a systemically administered drug and only apply the tumor harming treatment locally to the affected tissue. Radiation therapy can provide this local harm even though the radiation doses are delivered to all cells within the irradiation area. Nevertheless, radiation therapy remains an important curative treatment modality, with more than 50% of the patients benefiting from this treatment (Barton et al., 2006; Chen and Kuo, 2017). In principle, high-energy radiation is used to destroy cancer cells by depositing energy that damages the genetic material and prevents further cell proliferation. The type of radiation and the chosen method for delivering the energy to the malignant tissue is highly dependent on the type of tumor. Radiation can mainly be divided into two classes: X-rays and gamma-rays belong to the group of electromagnetic radiation whereas electron, neutron, proton and hole atom irradiation can be assigned to particle radiation (Gianfaldoni et al., 2017). Even though radiation therapy is often used in combination with surgery and chemotherapeutic modalities to increase the anti-tumor effect, long term cancer survivors have a higher risk for developing second malignancies because the radiation does also harm genetic material of healthy tissue (Dracham et al., 2018). Thus, the delivered dose has to be as low as possible to prevent harm to healthy tissue but it has to be effective enough to eliminate cancer cells. Various approaches were developed to increase the radiation sensitivity of tumor cells, but all of them require the administration of drugs, which selectively seek into cancer cells. This can be achieved by exploiting biochemical characteristics, which mark cancer cells differently from healthy tissues (Hanahan and Weinberg, 2011). For example, the dysregulation of translational regulators (Bhat et al., 2015), altered epigenetic regulation mechanisms (Pfister and Ashworth, 2017), the overproduction of enzymes (DeBerardinis and Chandel, 2016) or changes in the cellular microenvironment such as a lower pH (Kato et al., 2013) can be facilitated for selective therapeutic drug delivery. Moreover, cancer cells frequently overexpress cell surface proteins, which bind ligands of different nature, allowing the targeting of tumor cells by various molecule classes (Vhora et al., 2014).

The idea of targeted therapy was envisioned over 100 years ago by the German scientist Paul Ehrlich, who was awarded with the Nobel Prize in Medicine in 1908. Originally, he proposed the concept of specifically killing pathogens, which cause diseases in the body, without harming the body itself. Ehrlich named the hypothetical agent, which could facilitate this “magic bullet” (Ehrlich et al., 1956). His idea evolved over the time and his concept was transferred into cancer therapy where malignant cells have to be eliminated. Therefore, apoptosis inducing agents are combined with molecules, which selectively bind tumor cells. Although this concept seems to be quite simple, the fine-tuning is fairly elaborate and still challenging nowadays.

**Figure 1:**
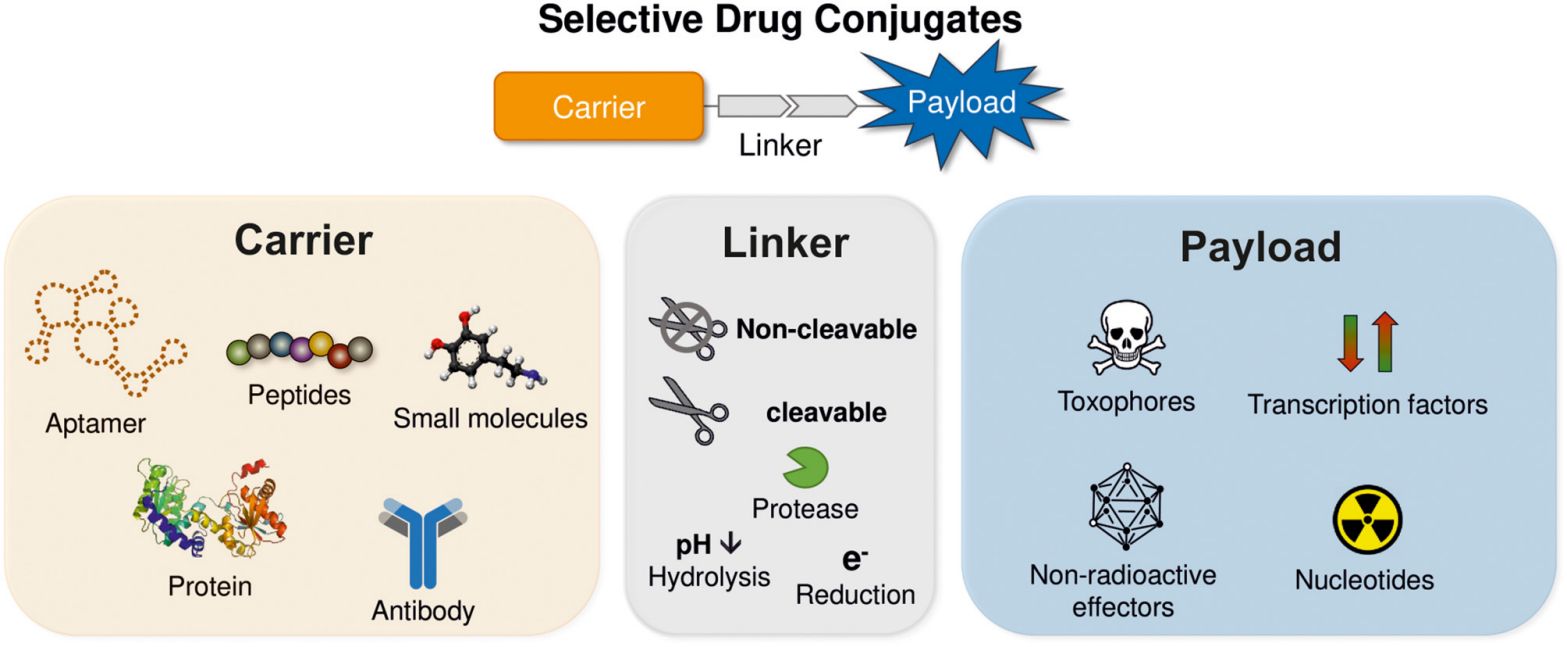
Schematic structure of receptor targeting drug conjugates. The drug conjugates are comprised of three modules: payload, linker, and carrier.

Various approaches have been investigated to achieve a selective delivery of different effector molecules to cancer cells. In principle, all conjugates used for selective drug delivery consist of three modules (Figure 1). The first component is the carrier, which facilitates tumor targeting. Besides aptamers also small molecules and biologics such as peptides, proteins and antibodies have been investigated intensively to facilitate sufficient tumor selectivity (Yoo et al., 2019). The second component is the drug itself, which can induce a variety of biological functions, but in context of cancer treatment mostly cytotoxic molecules or radionuclides are used. The third module links the former two. In dependence of the used payload, either cleavable or non-cleavable linkers can be applied to enable a controlled drug release, if necessary. Since all three components contribute to the overall biological efficacy and selectivity, they are described separately in more detail in the following sections.

## Tumor-Homing Agents: Antibodies vs. Peptides

The first component is the tumor-homing molecule. Multiple classes of molecules were investigated, including small molecules (Fitzgerald et al., 2016; Kue et al., 2016; Fernández et al., 2018), aptamers (Kaur et al., 2018; Kim et al., 2018), and polysaccharides (Choi et al., 2011). Even though these molecule classes led to promising results in preclinical studies, monoclonal antibodies (mAbs) evolved as fastest-growing drug class, with more than 30 mAbs being approved and more than 100 in clinical development over the past years (Carter and Lazar, 2018). So far, five antibody-drug conjugates (ADC) have received regulatory approval of the US Food and Drug Administration (FDA) and the European Medicines Agency (EMA) (Chau et al., 2019). All of them bind to cell surface receptors such as the CD22, CD30, CD33 or the human epidermal growth factor receptor 2 (HER2), which internalizes together with the ADC. After degradation of the ADC in the lysosome, the attached cytotoxic payload is released and the desired effect is induced. Although these conjugates reached the market and proved the feasibility of this approach, antibodies have serious limitations. Most mAbs do not penetrate into tumors. With a molecular weight of about 150 kDa, they are too big to diffuse efficiently into malignant tissue (Dreher et al., 2006; Jain and Stylianopoulos, 2010). Monoclonal antibodies can be immunogenic, even when they have been humanized and they tend to aggregate in excretory organs like liver and kidneys (Borsi et al., 2002; van Schouwenburg et al., 2010; Carrasco-Triguero et al., 2013). Moreover, the generation of mAbs is very expensive as well as time-consuming and non-selective payload conjugation can lead to reduced product homogeneity (Nejadmoghaddam et al., 2019).

Most of these drawbacks can be eliminated by using smaller biomolecules like peptides. In fact, peptide-drug conjugates (PDC) comprise several advantages as carrier molecules. Peptides with up to 50 amino acids can be easily synthesized in large scale to a reasonable price and modifications as non-natural amino acids can be directly introduced in the synthesis process (Firer and Gellerman, 2012; Mäde et al., 2014). The straightforward synthesis allows a rational optimization of side chains and backbone structures, which can result in increased binding affinities and physicochemical properties can be directly influenced (Erak et al., 2018). Furthermore, peptides are considered to be rapidly cleaved by proteolytic enzymes and quickly cleared from the blood circulation by liver and kidney. Those pharmacodynamic properties can be modulated by different modification and stabilization approaches (Vlieghe et al., 2010). One of the most known concepts of peptide stabilization is lipidation, which involves the incorporation of fatty acids into the peptide (Zhang and Bulaj, 2012). Fatty acids bind to serum albumin, preventing proteolytic cleavage in the blood by proteases and leads thereby to a prolonged circulation time (Frokjaer and Otzen, 2005). The long-acting glucagon-like peptide-1 (GLP-1) receptor agonists liraglutide (Victoza®) (Guryanov et al., 2016) and semaglutide (Ozempic®) (Marso et al., 2016), which are used to treat type-2 diabetes and obesity, are recent examples for this approach. Peptides are generally considered safe, since they feature low immunogenicity and produce non-toxic metabolites (Ahrens et al., 2012). Furthermore, their low molecular weight leads to an enhanced penetration into solid tissues resulting in better anti-tumor effects (Firer and Gellerman, 2012; Hock et al., 2015).

Peptides used in PDCs can be divided into two categories: cell-penetrating peptides (CPPs) and cell-targeting peptides (CTPs). The uptake mechanisms of CPPs across the cell membrane are not fully understood yet. Some CPPs are reported to cross the cell membrane by an energy-dependent cellular process like endocytosis- or receptor-mediated uptake, whereas others use energy-independent non-endocytic translocation pathways (Derossi et al., 1996; Thorén et al., 2005). Nevertheless, a significant increase in drug delivery was reported for small toxophores as well as for proteins being attached to CPPs like the TAT peptide, Pep-1 or Transportan (Morris et al., 2001; Muratovska and Eccles, 2004; Duong and Yung, 2013). However, extensive application of CPPs is limited due to its low cell specificity (Regberg et al., 2012).

In contrast, CTPs are ideal carrier molecules as they possess the same ability as mAbs. They bind with high affinity to overexpressed receptors on the tumor cell surface without exhibiting the disadvantages of mAbs. However, the conjugation of payloads to the peptide molecules is more critical because receptor binding and selectivity can be affected due to the steric demand of the payload, which can interfere with receptor recognition. Therefore, extensive knowledge of the interaction between peptide and receptor is needed to introduce drug cargos rationally. Amino acids like lysine, cysteine, glutamate, serine, which are not involved in the receptor recognition, can be used directly for payload conjugation at the sidechain or not required positions can be exchanged by those to introduce possible modification sites (Mäde et al., 2014). Many peptides also allow simple N-terminal modification because their N-terminus is not involved in the receptor recognition. Ideally, peptide carriers contain multiple modification sides, which allow the incorporation of multiple payloads per peptide molecule. The increased payload loading can enhance the therapeutic effects due to the increased drug concentrations at the tumor site (Dubowchik et al., 2002; Böhme et al., 2016).

## Peptide Receptors as Targets in Cancer Therapy

A major challenge in the development of novel and highly effective anti-cancer drugs is the selective drug-delivery to the tumor site while healthy tissue is spared. Cell surface receptors are of high interest in the targeted cancer therapy approach as they provide the desired properties to allow selective tumor targeting (Table 1) (Reubi, 2003; Vhora et al., 2014). One of these requirements is the ectopical overexpression in high amounts on the malignant tissue to facilitate a sufficient selectivity. A tumor-to-normal-cell expression ratio of 3:1 or higher is usually desired. Secondly, the amounts of overexpressed receptors have to be sufficient to ensure drug delivery in appropriate amounts to obtain the desired therapeutic effect (Reubi, 2003; Vrettos et al., 2018). The determination of these expression levels is not trivial since tissue sampling and processing is difficult to standardize. Expression levels can vary not only within biopsies, but also over the time course of the therapy and therefore each treatment plan has to be tailored for every patient.

**Table 1:** Overview of peptide-binding receptors studied for anti-cancer drug delivery.

**Targeted receptor**	**Tumor expression**	**References**
Integrin (αvβ3)	Activated endothelial cells and tumor cells (such as U87MG glioblastoma cells), ovarian cancer cells	Desgrosellier and Cheresh, 2010; Chen and Chen, 2011
EGFR	Lung, breast, bladder, and ovarian cancers	Li et al., 2005; Yarden and Pines, 2012
NPY (hY1R)	Breast cancer, Ewing sarcoma	Söll et al., 2001; Körner et al., 2008; Li et al., 2015
Bn receptors (GRPR)	Lung, prostate, breast, pancreatic, head/neck, colon, uterine, ovarian, renal cell, glioblastomas, neuroblastomas, gastrointestinal carcinoids, intestinal carcinoids, and bronchial carcinoids	Jensen et al., 2008; Sancho et al., 2011
Somatostatin (SSTR2)	Small cell lung, neuroendocrine tumor, prostate cancer, breast cancer, colorectal carcinoma, gastric cancer, hepatocellular carcinoma	Volante et al., 2008; Sun and Coy, 2011
GnHR-R	Ovarian, breast, prostate, lung cancer	Schally and Nagy, 1999; Limonta et al., 2012
VIP receptors	Endocrine tumors, colon, breast cancer	Reubi, 2003; Tang et al., 2014
MC1R	Melanoma tissues	Froidevaux and Eberle, 2002; Froidevaux et al., 2005
Neurotensin receptors (NTSR1)	Breast, colon, pancreatic, lung, prostate cancer	Kokko et al., 2003; Wu et al., 2012

Even though two peptide-drug conjugates have already been approved by the FDA for treatment and diagnosis of cancer, the development of novel peptide-drug conjugates targeting overexpressed receptors is still very challenging. In the following section, comprehensively studied peptide binding receptors will be presented, which are considered as promising targets in targeted cancer therapy.

### Integrins

A frequently used peptide carrier is the tripeptide motif arginine-glycine-aspartic acid (RGD), which was identified in the 1980s within fibronectin that was known to bind integrins (Pierschbacher and Ruoslahti, 1984; Plow et al., 2000; Kapp et al., 2017). The membrane-bound receptor family appears as heterodimers of non-covalently connected α- and β-subunits. Integrins link the extracellular matrix with the intracellular cytoskeleton to mediate cell adhesion, migration, and proliferation (Hynes, 1987; Schwartz et al., 1995). Since all of these processes are highly relevant for carcinogenesis, the integrin αvβ3 evolved as important anti-cancer target primarily because it is associated with tumor angiogenesis and metastasis (Cox et al., 2010). It is overexpressed also in activated endothelial cells, new-born vessels, and various other tumor cells but is not found in most adult epithelial cells (Cai and Chen, 2006; Desgrosellier and Cheresh, 2010).

Targeting of these cancer types is mostly accomplished by using the cyclic RGD variant c(RGDfK) owing to its improved affinity for the integrin receptors (Ryppa et al., 2008; Chen and Chen, 2011; Gilad et al., 2016a). Recently, internalizing RGD (iRGD) conjugates gained considerable attention due to its improved vascular and tumor-tissue permeability (Sugahara et al., 2009). The sequence CRGDKGPDC, in which the cysteines at the C- and N-terminus are connected through a disulfide bond, allowed a proteolytic cleavage after integrin binding. The cleaved peptide gains affinity for neuropilin-1 (NRP-1), which is then involved in a not fully understood internalization mechanism (Sugahara et al., 2009; Kadonosono et al., 2015). However, using this peptide as carrier allows the generation of smart delivery vehicles that facilitate an active and directed uptake of the drug into tumor cells (Cho et al., 2016).

### Epidermal Growth Factor Receptor (EGFR)

Another highly interesting transmembrane protein is the epidermal growth factor receptor (EGFR), which belongs to the ErbB family. Apart from EGFR/HER1 (ErbB-1), HER2/neu (ErbB-2), HER3 (ErbB-3), and HER4 (ErbB-4) belong also to this tyrosine kinase family (Wieduwilt and Moasser, 2008). EGFR was found to be overexpressed in very high amounts in various cancer types and is associated with a strongly enhanced proliferation rate of cancer cells (Yarden and Pines, 2012). Therefore, many monoclonal antibodies and small molecule inhibitors target the EGFR in a clinical setting, inhibiting EGFR and decelerating tumor growth (Seshacharyulu et al., 2012).

EGFR is also considered as a uptake system for targeted drug delivery owing to its internalization behavior upon ligand binding and receptor dimerization (Grandal and Madshus, 2008). Several short peptides have been identified by phage display libraries (Li et al., 2005; Ai et al., 2013). For example, the GE11 peptide (YHWYGYTPQNVI) was conjugated to liposomes (Zou et al., 2018), nanoparticles (Li et al., 2019), or toxophores like doxorubicin (Fang et al., 2017).

### G Protein-Coupled Receptors (GPCRs)

G protein-coupled receptors form the biggest class of transmembrane proteins with more than 800 receptors. Approximately 15% of these are targeted by ca. 35% of all FDA approved drugs, demonstrating the tremendous pharmacological potential of this receptor class (Sriram and Insel, 2018). GPCRs share the common structure of seven transmembrane (TM) helices, which are linked by three intra- and three extracellular loop regions, an extracellular N-terminus, and an intracellular carboxyl-terminal domain (Rosenbaum et al., 2009). Classical GPCR signaling is initiated by an extracellular ligand binding, which induces a conformational change in the receptor that initiates binding and activation of intracellular heterotrimeric G proteins (Gα_i_, Gα_q_, Gα_s_) (Weis and Kobilka, 2018). The physiological response depends strongly on the activated G protein. Since the induced signaling cascades control elementary processes within the cell, malfunctions in this system are often associated with cancerogenesis (Nieto Gutierrez and McDonald, 2018). In these cases, GPCRs are frequently found to be overexpressed, allowing the specific targeting of tumor cells with peptide-drug conjugates (Insel et al., 2018).

**Figure 2:**
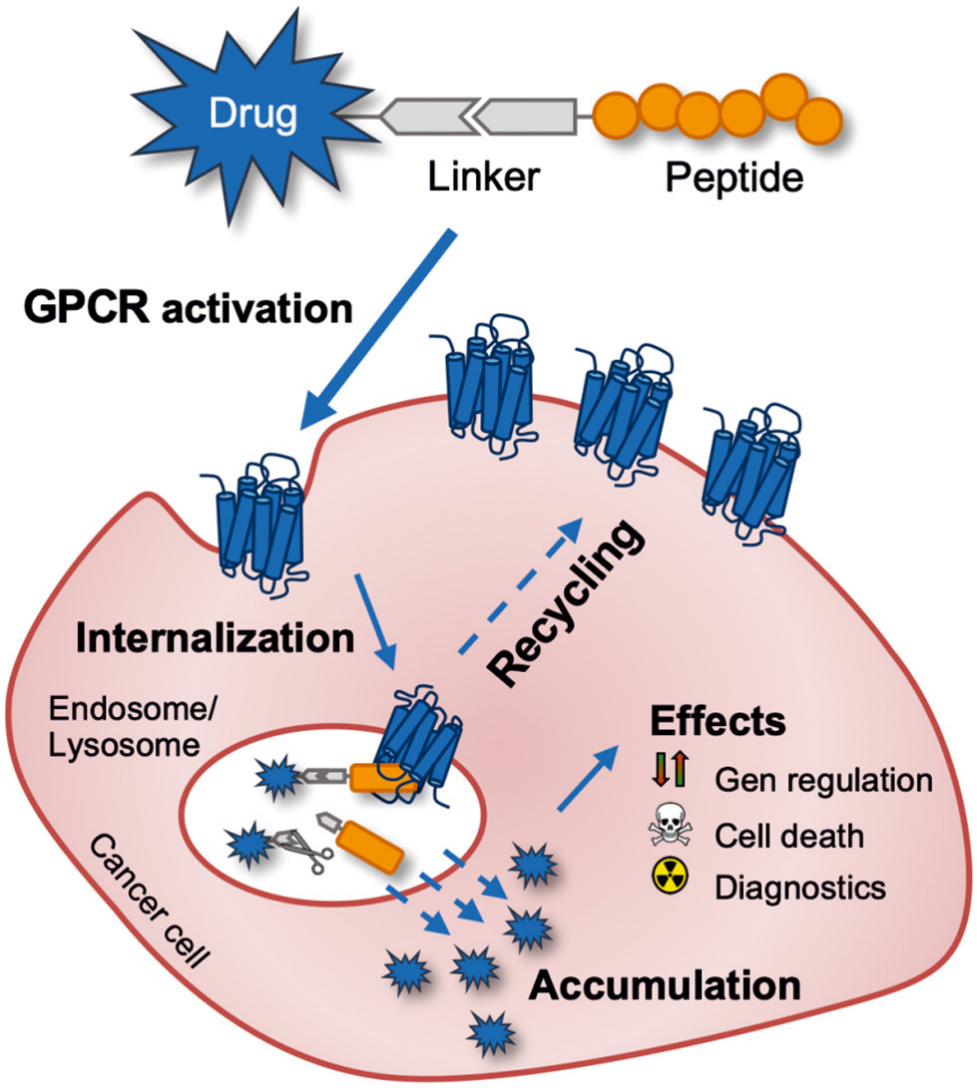
Schematic outline of targeting a tumor-expressed G protein-coupled receptor for anti-cancer drug delivery with a peptide-drug conjugate. The drug can be released intracellularly by intentionally using a cleavable linker or just by endo-lysosomal degradation of the peptide-drug conjugate.

Besides the overexpression, GPCRs are known to desensitize after activation by intracellular phosphorylation mediated by GPCR kinases (GRKs). The subsequent recruitment of adaptor proteins such as arrestin induces the clathrin-mediated endocytosis (Moore et al., 2007). The entire receptor-ligand complex is internalized in this process and translocated to the lysosome. Some GPCRs are separated from the ligand in the endosome and recycle back to the cell membrane, where they can be reactivated and internalized. This mechanism can be exploited to actively accumulate drug molecules inside the cell making peptide-drug conjugates a highly interesting and suitable class of molecules as a cellular drug delivery system (Figure 2) (Vrettos et al., 2018). In this review, the gastrin-releasing peptide receptor and the human Y_1_ receptor will be presented in more detail.

#### NPY Receptor Family

**Figure 3:**
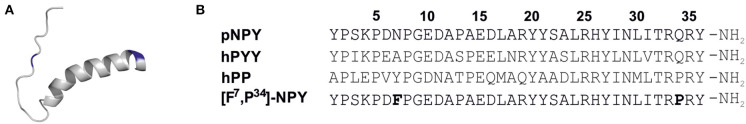
**(A)** Three-dimensional solution structure of human NPY determined by nuclear magnetic resonance spectroscopy (PDB: 1RON). Substituted amino acids in [F^7^,P^34^]NPY are indicated in blue. **(B)** Amino acid sequences of pNPY, hPYY, hPP, and the hY_1_R-preferring [F^7^,P^34^]-NPY.

The human Y_1_ receptor (hY_1_R) is a member of the neuropeptide Y (NPY) receptor family, which is comprised of three additional receptors, namely the Y_2_ receptor (hY_2_R), the Y_4_ receptor (hY_4_R), and the Y_5_ receptor (hY_5_R) (Michel et al., 1998). The human Y receptors (hYR) are recognized and activated by the neuropeptide Y peptide hormone family including the 36-amino-acid NPY, peptide YY (PYY), and pancreatic polypeptide (PP) (Figure 3). All native peptides are composed of a flexible N-terminus, a C-terminal amphipathic α-helix and an amidated C-terminus (Larhammar, 1996; Monks et al., 1996). The Y receptor/NPY hormone family forms a multi-ligand/multi-receptor system in which NPY and PYY display high affinity for the hY_1_R, hY_2_R, and hY_5_R, whereas PP has the highest affinity for the hY_4_R (Pedragosa-Badia et al., 2013). However, all three peptides bind and activate all four receptors to some extent. While NPY is mainly expressed in the central nervous system (CNS) where it acts as a neurotransmitter regulating energy homeostasis and anxiety (Colmers and Bleakman, 1994; Wettstein et al., 1995), PYY and PP were found in peripheral tissues acting as hormones regulating the vasoconstriction (Michel and Rascher, 1995), insulin release (Wang et al., 1994), and gastrointestinal and renal epithelial secretion (Playford and Cox, 1996). The association with metabolic diseases like diabetes, obesity, hypertension, and dyslipidemia led to the development of various selective small molecules, which bind and activate only one of the Y receptors (Yulyaningsih et al., 2011; Tan et al., 2018; Yi et al., 2018). hY_1_R-selective molecules were particularly interesting, not only because inhibition of the hY_1_R leads to an anorexic effect (Brothers and Wahlestedt, 2010) but also because the hY_1_R was found to be present in certain tumor tissues. While ovarian sex cord-stromal tumors, nephroblastomas, gastrointestinal stromal tumors and testicular tumors were found to express the hY_1_R in combination with the hY_2_R, adrenal cortical tumors, Ewing sarcoma tumors, and renal cell carcinomas display exclusive hY_1_R expression (Körner and Reubi, 2007; Körner et al., 2008).

An outstanding hY_1_R expression profile was identified in breast cancer by Reubi et al. (2001). He demonstrated that the hY_1_R is expressed in very high density in 85% of investigated primary human breast tumors and in 100% of breast cancer-derived metastases (Reubi et al., 2001). In contrast to the previously described tumors, the non-neoplastic breast tissues showed no hY_1_R expression. Instead, the hY_2_R was predominantly found in non-malignant tissue. This switch in the expression profile from hY_2_R in healthy tissue to hY_1_R in breast tumors allows the selective targeting of these cancer cells if a hY_1_R-preferring ligand is used (Figure 3).

The use of selective agonists is favored because agonists exploit the desensitization mechanism of the Y-receptors to deliver certain payloads to intracellular compartments. All Y receptors desensitize by clathrin-mediated endocytosis in complex with the bound ligand after activation. While the internalization process for the hY_5_R is relatively slow, the other Y-receptors internalize rapidly within few minutes (Gicquiaux et al., 2002). The recycling of the hY_1_R and hY_2_R back to the cell membrane has been observed by fast and slow endosomal routes (Gicquiaux et al., 2002; Mörl and Beck-Sickinger, 2015). These recycling processes are essential for an active drug accumulation inside the cell.

In order to use this drug shuttle system, many studies focused on the development of hY_1_R preferring agonists, which turned out to be challenging due to the great similarities between the receptors and native peptides (Zhang et al., 2011). Nevertheless, the shortened, C-terminally derived analog [Pro^30^, Nle^31^, Bpa^32^, Leu^34^]NPY(28-36) was identified as a hY_1_R-selective agonist in 2009 (Zwanziger et al., 2009). Another hY_1_R-selective agonist is the full length NPY analog [F^7^,P^34^]-NPY, which exhibits nanomolar potency at the hY_1_R and a highly reduced affinity for the hY_2_R (Söll et al., 2001). This derivative was already used in *in vivo* studies and serves as a first proof of concept for this receptor targeting approach. A fluorine-18 (^18^F)-labeled, fluoroglycosylated [F^7^,P^34^]-NPY analog was synthesized and enabled the visualization of hY_1_R-expressing MCF-7 tumor cells in a xenograft mice model (Hofmann et al., 2015). Furthermore, four breast tumor patients received a technetium-99m labeled [F^7^,P^34^]-NPY conjugate. While no significant peptide uptake was observed in healthy volunteers, the primary tumor in all four patients as well as the metastatic sites were clearly visualized by whole-body scintimammography (Khan et al., 2010). These studies demonstrated the tremendous potential of the hY_1_R as a target in a selective drug delivery system for breast cancer.

#### Bombesin Receptor Family

The mammalian bombesin (Bn) receptor family consists of three GPCRs: the neuromedin B (NMB) receptor (NMBR or BB1-receptor), the gastrin-releasing peptide (GRP) receptor (GRPR or BB2-receptor) and the orphan bombesin receptor subtype 3 (BRS-3 or BB3-receptor) (Jensen et al., 2008). All three receptors are widely expressed in the CNS where they are associated with processes including satiety, thermoregulation, stress and fear responses (Roesler et al., 2006; González et al., 2008). They are also found in the gastrointestinal tract, where they are mainly involved in smooth muscle contraction and gastrin release (Uehara et al., 2011).

These receptors form together with their natural ligands a multi-ligand/multi-receptor system. While NMB binds with high affinity to the NMBR, GRP prefers the GRPR. The endogenous ligand of the BRS-3 could not be identified so far. Nevertheless, all three receptors are combined in one family because the *amphibian* 14-mer peptide homolog Bn (Sequence: Pyr-QRLGNQWAVGHLM-NH_2_), which was isolated from the skin of the European fire-bellied toad, binds and activates all three receptors (Anastasi et al., 1971; Erspamer et al., 1972). All bombesin-like peptides share two common features: their C-terminus is amidated and the last seven C-terminal amino acids are highly similar (McDonald et al., 1979; Erspamer, 1988; Kroog et al., 1995; Hellmich et al., 1997). The Bn receptors (BnR), especially the GRPR, have been extensively studied and found to be overexpressed in several human cancers including breast, colon, non-small cell lung cancer, gliomas, meningiomas, head/neck squamous cell, ovarian, pancreatic, and prostate cancers, and neuroblastomas (Table 2) (Gugger and Reubi, 1999; Markwalder and Reubi, 1999; Reubi et al., 2002a,b; Moody et al., 2004; Pu et al., 2015; Moreno et al., 2016). Since the BnR are expressed in a number of common tumors, increasing interest rose not only to target the BnR for tumor localization and visualization but also to deliver cytotoxic agents (Schroeder et al., 2009; Sancho et al., 2011; Yu et al., 2013).

**Table 2:** Incidence of bombesin receptor subtype expression in various human cancers (Reubi et al., 2002b).

**Tumor type**	***n* cases**	**Receptor incidence**		
		**NMBR**	**GRPR**	**BRS-3**
Prostate carcinomas	12	0/12	12/12	0/12
Breast carcinomas	57	0/57	41/57	0/57
**NE**^**a**^ **GEP tumors**				
Gastrinomas	5	0/5	5/5	0/5
Intestinal carcinoids	24	11/24	0/24	0/24
Thymic carcinoid	1	1/1	0/1	0/1
**NE lung tumors**				
Bronchial carcinoids	26	1/26	0/26	9/26
Small cell lung cancers	9	0/9	3/9	4/9
LCNEC	1	0/1	0/1	1/1
Renal cell carcinomas	16	0/16	6/16	4/16
Ewing sarcomas	10	0/10	0/10	2/10

a *NE, neuroendocrine; GEP, gastroenteropancreatic; LCNEC, large cell neuroendocrine carcinoma*.

More than 200 reports were published focusing on the investigation of various Bn-like peptides conjugated primarily with radionuclides, including ^9m^Tc, ^111^In, ^67^Ga, ^68^Ga, ^64^Cu, ^177^Lu, ^90^Y or ^213^Bi, which were either used for tumor diagnostic or peptide receptor radionuclide therapy (PRRT) (Dash et al., 2015).

**Figure 4:**
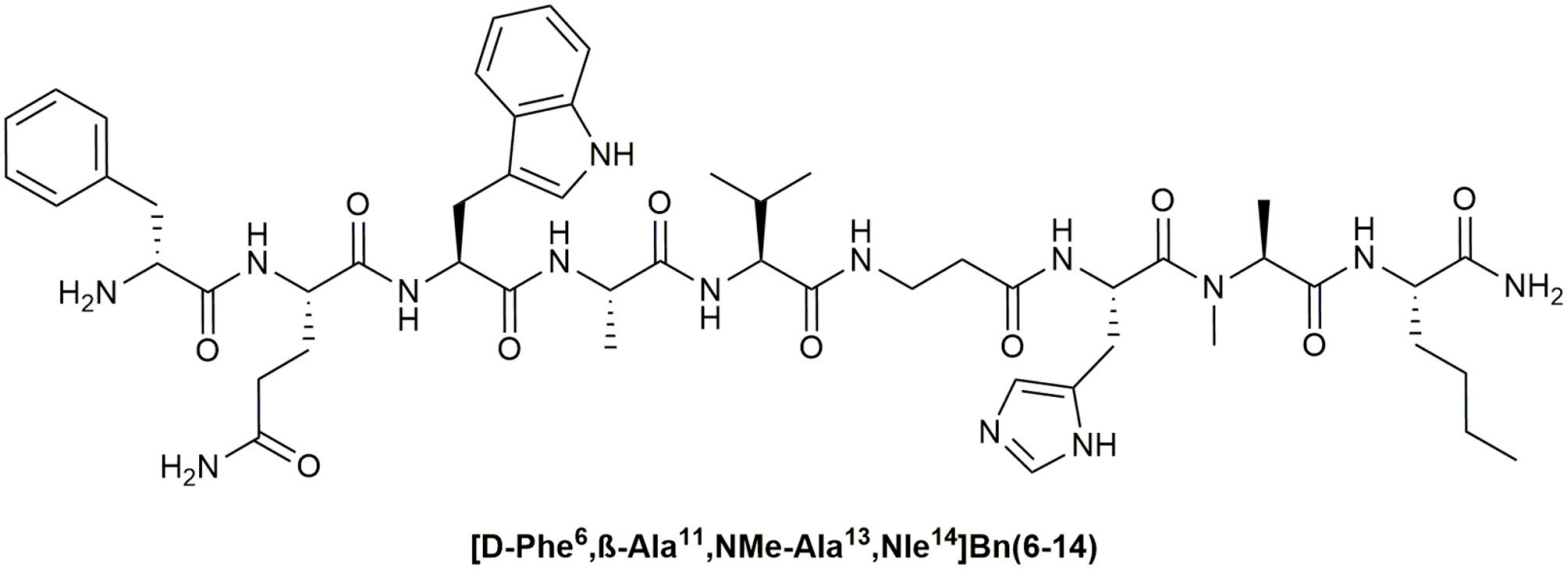
Chemical structure of a stable GRPR-selective agonist.

A number of these studies reported excellent visualization of BnR overexpressing tumors like prostate cancer *in vivo* as well as in humans (Scopinaro et al., 2002, 2004; van Essen et al., 2009). Thereby, radiolabeled BnR antagonists were found to be more suitable for tumor visualization applications then BnR agonists because they showed higher tumor uptake and better imaging properties (Ginj et al., 2006; Cescato et al., 2008; Mansi et al., 2013). This might be explained by better plasma stability of BnR antagonists compared to agonists, and their higher selectivity for the GRPR. In many studies the synthetic Bn peptide agonist [d-Phe^6^, β-Ala^11^, Phe^13^, Nle^14^]Bn(6–14) and its d-Tyr^6^ analog were used due to their high affinity for the GRPR (Mantey et al., 1997; La Bella et al., 2002; Schroeder et al., 2009). However, the NMBR and the BRS-3 were bound with similar potencies (IC_50_, 0.3–2 nM) leading to off-target effects and reduced effective concentrations at the tumor side (La Bella et al., 2002). Thus, the potential tumor uptake is theoretically lower in comparison to a stable antagonist, which features comparable binding properties. Moreover, the rapid degradation of common Bn agonists in blood plasma reduces the potential uptake by tumor cells even further (Bläuenstein et al., 2004). A stable and selective GRPR-agonist could potentially feature similar or even better tumor uptake values compared to antagonists. Nevertheless, the development of GRPR-selective peptide agonists, which feature sufficient plasma stability is still challenging and was addressed only in few studies (Darker et al., 2001; Valverde et al., 2013). Recently, we could identify the peptide [d-Phe^6^, β-Ala^11^, NMe-Ala^13^, Nle^14^]Bn(6–14), which displays high activity at the GRPR, more than 4,000-fold selectivity and > 75% blood plasma stability after 24 h (Hoppenz et al., 2019) (Figure 4).

By using agonistic Bn peptides, the desensitization mechanism of the GRPR can be exploited for an active drug shuttling into the tumor cells. Following the clathrin-mediated internalization, the GRPR is separated from the endosome and recycles back to the cell membrane rapidly, where it can be activated again (Slice et al., 1998). Based on this approach, different bombesin conjugates were synthesized bearing toxophores (Stangelberger et al., 2006; Moody et al., 2007), siRNA (Rellinger et al., 2015), nanoparticles (Jafari et al., 2015), or liposomes (Accardo et al., 2012) demonstrating great results in preclinical and clinical studies.

#### Somatostatin Receptors (SSTRs)

Another very intensely studied receptor family is the somatostatin receptor (SSTR) family, which is comprised of five members (SSTR1–5). Their natural ligand is the disulfide-cyclized oligopeptide somatostatin (SST), which occurs in two active isoforms, the SST-14 and SST-28 (Reisine and Bell, 1995). Both of these isoforms bind and activate the SSTRs, which are widely expressed in different tissues in the body including nervous, pituitary, kidney, lung and immune cells (Patel, 1999). The interaction of SST and its receptors not only controls the endocrine system and neurotransmission but also provides potent antisecretory and antiproliferative activities (Patel, 1999; Olias et al., 2004). Owing to these features, metabolically stabilized derivatives of somatostatin including octreotide (Sandostatin®), lanreotide, or pasireotide are directly used to treat growth hormone-producing tumors (Keskin and Yalcin, 2013). The SSTR2 and SSTR5 are thereby primarily targeted because they have been found in various neuroendocrine tumors as well as other tumors such as breast, ovarian and lung cancer (Reubi et al., 1992; Volante et al., 2008). Besides the direct targeting of the SSTRs, peptide-drug conjugates were intensely studied to even further boost the antiproliferative effect of these peptides. For example, Paclitaxel (Shen et al., 2008), Doxorubicin (Nagy et al., 1998), and Camptothecin (Sun et al., 2008) were conjugated to octreotide analogs and provided impressive toxicities and selectivity for SSTR2 and SSTR5 expressing cells *in vivo*. Furthermore, the conjugation of ^111^Indium to octreotide led to the first FDA approved peptide-drug conjugate (Octreoscan®), which can be used for diagnostic tumor imaging (Forssell-Aronsson et al., 2004).

### Other Receptors

In addition to the already introduced receptor targets, a number of other peptide receptors are also investigated as potential targets for anti-cancer drug delivery and will be covered briefly in this section.

The first example is the gonadotropin-releasing hormone receptor (GnRH-R), also known as luteinizing hormone-releasing hormone (LHRH) receptor, which is primarily expressed on gonadotrope cells in the pituitary but also found in lymphocytes, breast, ovary, and prostate (Harrison et al., 2004). The activation of the GnRH-R causes the release of follicle-stimulating hormone (FSH) and luteinizing hormone (LH), which are known as gonadotropins. Over the past decade, the GnRH-R emerged as promising drug delivery systems owing to its ectopic overexpression in a variety of human tumors such as prostate, endometrial, epithelial ovarian, bladder, breast, lymphomas, and lung cancers (Halmos et al., 2000; Keller et al., 2005; Gründker et al., 2011). This receptor can be addressed by agonistic peptides or small molecules in cancer therapy (Gründker and Emons, 2017). For the generation of drug conjugates, the selective GnRH analog [d-Lys^6^]-GnRH-I is frequently used and reached as a doxorubicin derivative (AEZS-108) clinical phase III (Kovács et al., 2007; Yu et al., 2017).

Another example are the vasoactive intestinal peptide (VIP) receptors 1 and 2, which were found to be overexpressed in colon, breast, and endocrine tumors. The natural ligand VIP and its analogs are considered to be a valuable target for the molecular imaging of tumors and therapeutic interventions (Moody et al., 2007; Tang et al., 2014).

The melanocortin receptor 1 (MC1R) is also a highly attractive target as it has been found to be highly expressed in the majority of melanomas (Miao and Quinn, 2008). Since traditional chemotherapy treatment of metastatic melanoma is not very effective, MC1R targeting peptide-drugs are highly desired (Helmbach et al., 2001). Apart from the natural ligand α-melanocyte-stimulating hormones (α-MSH), the short agonistic peptide NAPamide is used as a base for the development of potent peptide-drug conjugates (Froidevaux et al., 2005).

Furthermore, the neurotensin receptor 1 (NTSR1) was found to be overexpressed in breast, prostate, colorectal, lung, liver, and pancreas cancers among others. Since the metabolic stability of the natural ligand neurotensin was not sufficient for the use as peptide-drug conjugate, tetra-branched neurotensin peptides were synthesized and conjugated with various toxic agents such as 5-fluorodeoxyuridine (5-FdU), MTX and a chlorambucil alkylating agent (Falciani et al., 2010). Moreover, pseudo-neurotensin derivatives [Lys^8−9^]NT(8-13) were used as carrier molecules targeting the NTSR1 (Kokko et al., 2003; Gaviglio et al., 2012).

## Intelligent Linker Technologies

**Figure 5:**
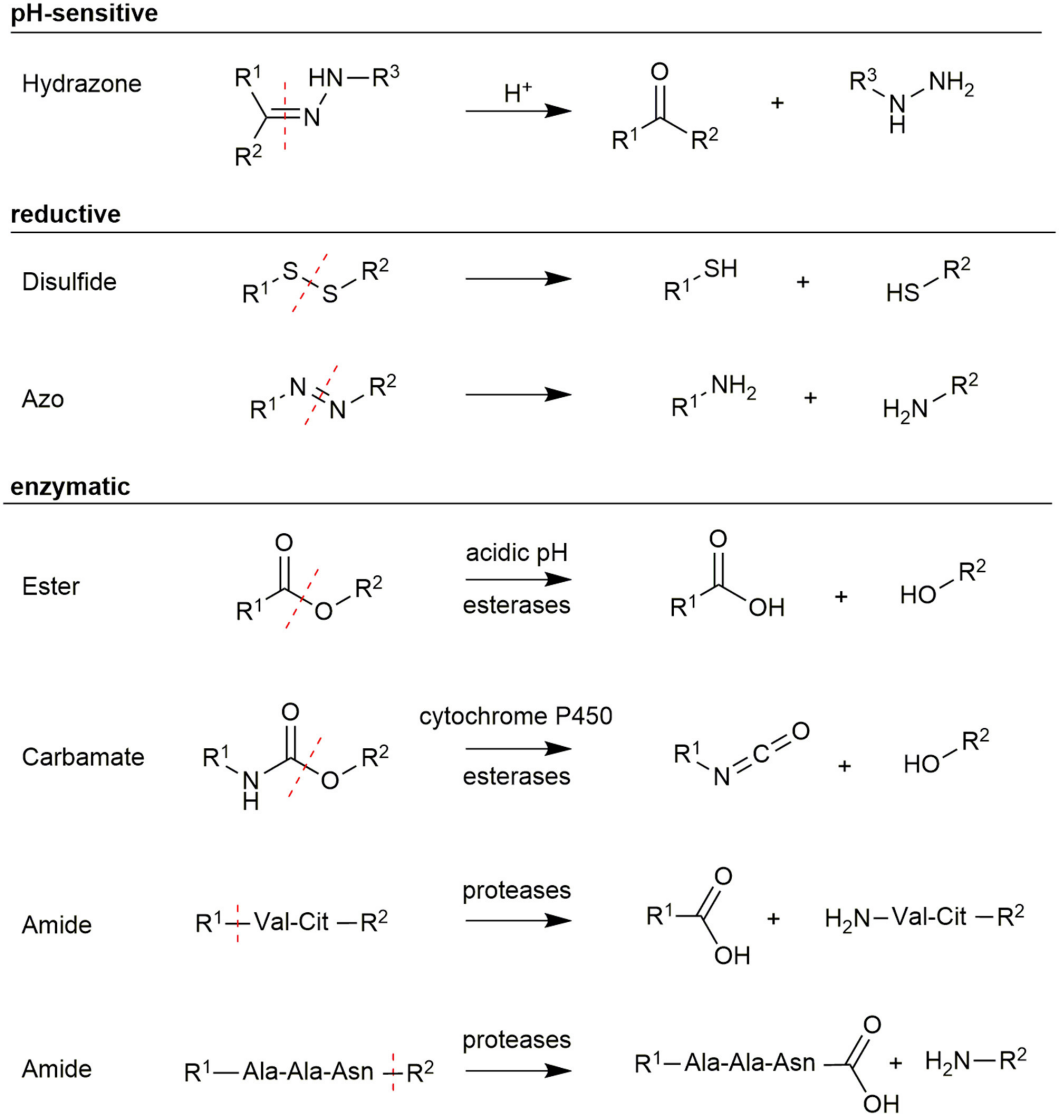
Overview of different linker structures used for payload conjugation and their cleavage mechanism.

Another very crucial aspect in the design of PDCs is the selected linker technology, which connects the carrier molecules and the payload. The linker structure has to provide distinct properties that ensure the greatest possible selectivity and biological efficacy of the payload (Figure 5). While, for example, radionuclides do not require the release after reaching the tumor cell, it is crucial for toxic payloads to be released to exploit their full potential. PDC prodrugs can fulfill this requirement by utilizing a linker structure, which controls the activity of the toxophore as long it is conjugated to the carrier peptide. As the tumor site is reached, the linker is selectively cleaved and the toxophore is released and thereby activated (Kratz et al., 2008). However, the linker must be stable during the circulation in the blood to avoid a premature release of the toxic agent, which would result in undesired peripheral toxicity. Different linker strategies have been reported for PDCs using various changes in the biological environment of cancer cells to facilitate the controlled release (Bildstein et al., 2011; Joubert et al., 2017).

One possibility to realize a selective release of toxophores is the use of pH-sensitive linkers. Since tumor cells grow and metabolize rapidly, tumor vessels are often unable to provide sufficient nutrients and oxygen. Anaerobic glycolysis inside the tumor cells produces lactic acid, which causes an acidification of the tumor tissue (pH 6.2–6.8) (Kato et al., 2013). A more significant difference in the pH-value was found in lysosomes, which features values between 4.5 and 6.0 (Ohkuma and Poole, 1978). Since agonistic PDCs are internalized into these compartments, a selective release of payloads can be achieved by using pH-sensitive linkers. The most frequently used pH-sensitive linker is a hydrazone bond, which is stable in neutral environments but easily hydrolyzed under weak acidic conditions. This concept has been demonstrated for antibodies (Ducry and Stump, 2010) as well as for peptides (Langer et al., 2001). Beside hydrazone bonds also imine, oxime, acetal, or cis-aconityl linkages were used (Chang et al., 2016).

Another approach uses redox-sensitive linkers. This is based on the high concentrations of antioxidants like glutathione (GSH) in the cytoplasm, which are 1,000-fold higher than in the plasma (15 mM intracellular vs. 15 μM extracellular) (Meister and Anderson, 1983). The concentration of GSH in cancer cells was found to be even higher due to hypoxia, which is caused by the abnormal blood flow in tumor tissue (Bansal and Simon, 2018). Therefore, disulfide bonds have been extensively used in targeted drug delivery (Wang et al., 2012). These bonds can be readily integrated in PDCs by reaction of a cysteine-containing peptide with a thiol-including toxic agent or a prodrug (Saito et al., 2003). Recently, thioesters and azo-bond derivatives have been investigated as cleavable linker structures (Chen et al., 2013; Medina et al., 2013).

An exquisite way to facilitate a selective linker cleavage is the use of structures, which can be cleaved by specific enzymes. For example esters and carbamates, which are either hydrolyzed by the low lysosomal pH or enzymatically by esterases and cytochrome P450 (Patterson et al., 1999; Cha et al., 2000). These structures can be introduced during peptide synthesis, but their extracellular stability needs to be carefully controlled because they are prone to hydrolysis in serum (Coin et al., 2007). However, great interest was gained by using short amino acid sequences as linkers, which are recognized explicitly by proteases being overexpressed in tumor tissues. Peptide bonds in these linkers are stable during circulation in plasma as the activity of the exploited protease is reduced due to the unfavorably high pH in plasma (Ciechanover, 2005). A wide range of short peptide sequences have been identified, including the short Val-Cit (Szlachcic et al., 2016) and Gly-Phe-Leu-Gly (GFLG) linkers (Naqvi et al., 2010), which are cleaved by the protease cathepsin B. Another very interesting protease is legumain because it is the only asparaginyl endopeptidase in mammals featuring very high substrate specificity for the amino acid sequence Ala-Ala-Asn (Chen et al., 1997; Dall and Brandstetter, 2016). The recognition sequence is cleaved C-terminally after Asn allowing a traceless release of the attached payload if the cargo is conjugated to the C-terminus, while the N-terminus of the linker is coupled to the carrier (Stern et al., 2009; Bajjuri et al., 2011; Mai et al., 2017). Therefore, enzymatically cleavable linkers are ideal structures to utilize a controlled release of toxic payloads within tumor cells especially because legumain and cathepsin B were found to be overexpressed in solid tumors (Liu et al., 2003; Gondi and Rao, 2013).

## Payloads in Targeted Cancer Therapy

The heart of every PDC is the drug cargo. While the peptide carrier and the linker structure provide the selectivity of the PDC, the drug is the component that facilitates the actual purpose of the conjugates. In many cases, the term “drug” refers to cytotoxic (chemotherapeutic) anti-cancer agents, but a broad spectrum of therapeutically active moieties can be meant. Conjugation of radionuclide complexes to peptides can result in molecules, which can be used for cancer diagnostics or PRRT. Moreover, non-radioactive molecules like boron can be used as cargo to generate PDCs for boron neutron capture therapy (BNCT).

### Chemotherapeutic Agents

**Figure 6:**
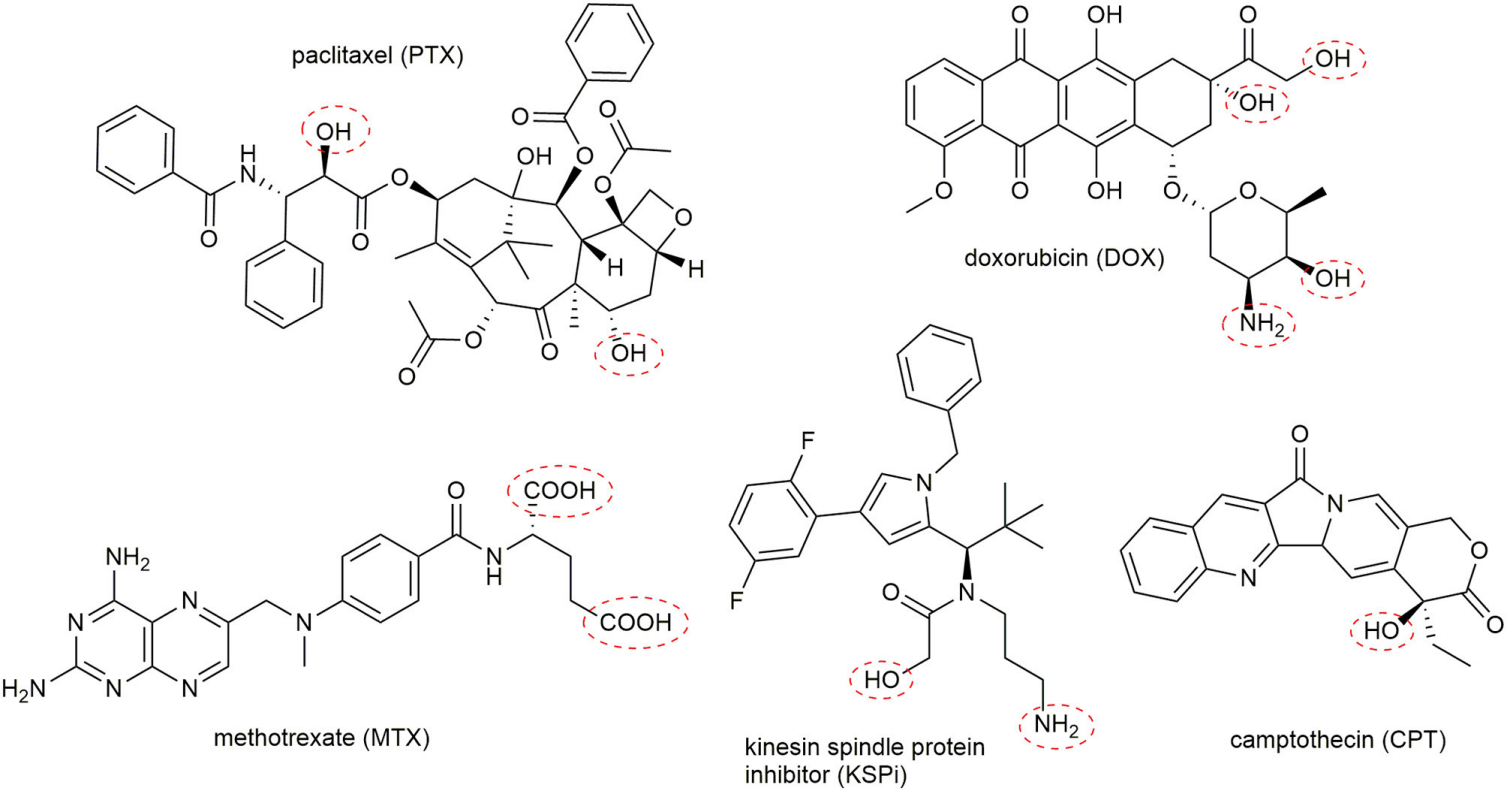
Representative examples of suitable toxic agents for the generation of peptide-drug conjugates. Frequently used conjugation sites in the toxophores are marked with red cycles.

Currently, more than 250 FDA approved drugs (Cancer.gov) are used to treat malignant cancers. Many of these classical chemotherapeutic drugs feature strong pharmacological activity owing to their great cytotoxicity. However, a disadvantage of these small molecules is their uncontrolled toxicity, which results in severe side effects. By attaching these molecules to targeting-moieties like peptides can enhance their pharmacokinetic and increase the therapeutic window of the parent cytotoxic agent (Chen et al., 2014). The selected drugs must comply with certain design principles in order to serve as suitable compounds for the generation of a PDC. Most importantly, intrinsic functional groups have to be available to enable the attachment to the peptide carrier by a cleavable linker. Fortunately, many toxic agents provide hydroxy, carboxy, or amine groups that can be used (Figure 6). In some cases, these groups are necessary for their biological response, enabling the generation of inactive prodrugs, which have to be released without a trace to facilitate their action at the tumor side (Kratz et al., 2008). Furthermore, the drug has to be chosen to exhibit sufficient and potent cytotoxicity vs. the malignant cells, as drug resistance mechanisms of cancer cells are frequently found (Housman et al., 2014).

**Figure 7:**
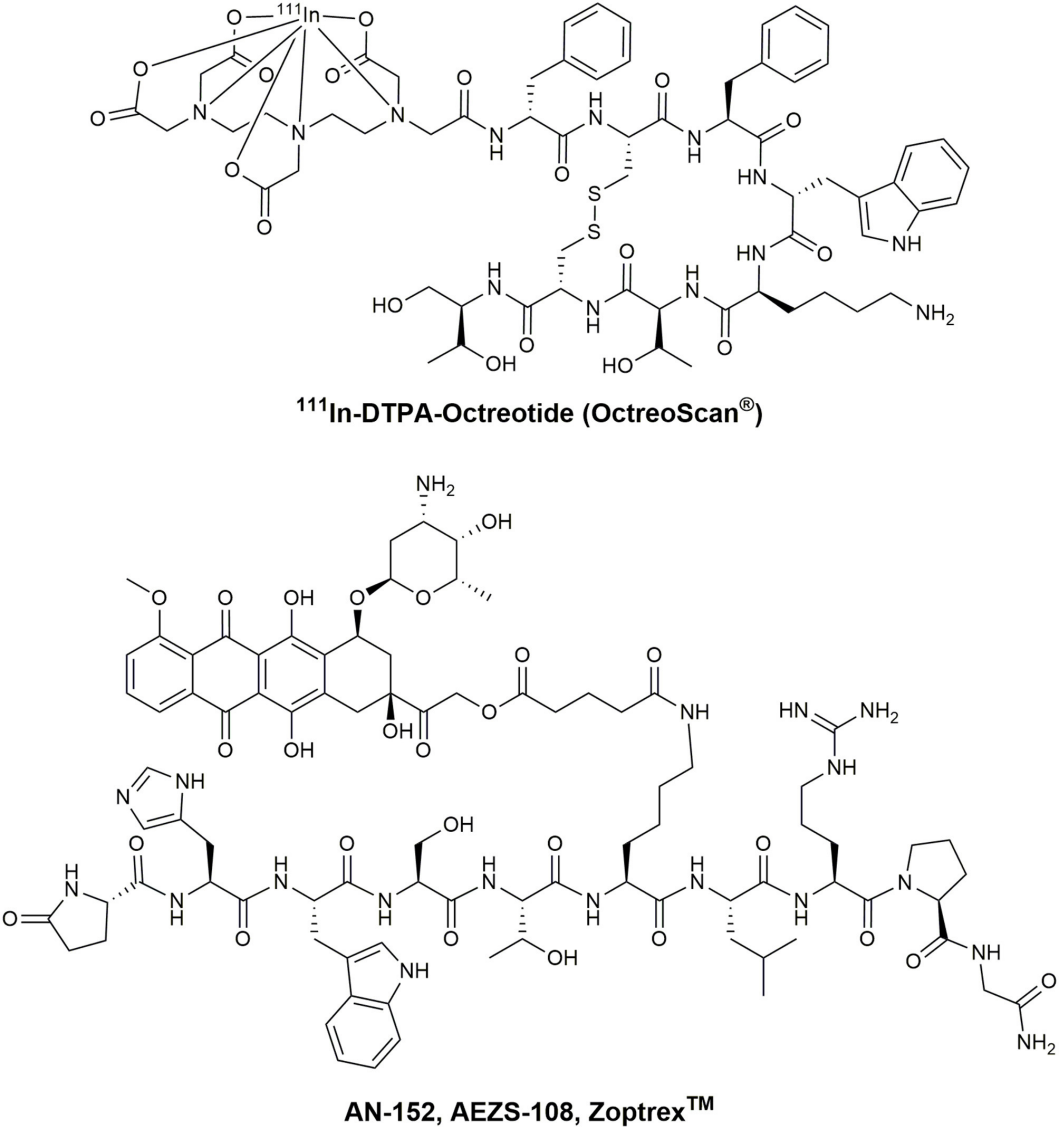
Chemical structure of OctreoScan® and Zoptrex™.

Chemotherapeutic agents used in PDCs can be classified by their general mode of action (Malhotra and Perry, 2003). The first group of molecules bind and interact with the cellular DNA or DNA-protein complexes. Thus, the transcription and DNA replication are disturbed, leading to the induction of apoptosis. Highly potent molecules including metal complexes (Weidmann et al., 2014), camptothecin (CPT) (Wall and Wani, 1996), and anthracyclines like daunorubicin (DAU) or doxorubicin (DOX) (Minotti et al., 2004) are broadly applied in PDCs (Figure 6). For example, the SSTR2 targeting octreotide was conjugated to DOX or 2-pyrrolino-DOX by ester linkers and exhibited selective toxicity against receptor-expressing tumors *in vivo* (Nagy et al., 1998; Huang et al., 2000; Engel et al., 2005; Shen et al., 2008; Seitz et al., 2009). Moreover, the integrin-targeting cyclic peptides c(RGDfK) and c(RGDfS) were conjugated to CPT and the alkylating agent chlorambucil by a carbamate and ester linkage, demonstrating growth inhibition in cancer cell lines expressing the integrin αvβ3 (Gilad et al., 2016b). In addition, CPT was coupled to the Bn analog [d-Tyr^6^,β-Ala^11^,d-Phe^13^,Nle^14^]Bn(6–14) by a carbamate linker exhibiting great cytotoxicity in Bn-receptor overexpressing cells while cells without these receptors were not affected (Moody et al., 2006). Until now, the most progressed receptor-targeting PDC for selective chemotherapy is Zoptarelin Doxorubicin (AN-152, AEZS-108, Zoptrex^TM^), which is composed of a GnRH analog and doxorubicin conjugated through an ester bond with a glutaric acid spacer (Figure 7) (Nagy et al., 1996). This derivative reached clinical phase 3 for the treatment of endometrial cancer, and the results were disclosed in May 2017. Even though very encouraging results were obtained before, Zoptarelin Doxorubicin neither extended overall survival nor improved the safety profile compared to the classical chemotherapy with DOX (ZoptEC, 2013).

The second class of frequently used toxophores convey their cytotoxicity by inhibiting the DNA-biosynthesis. These antimetabolites, for example the nucleoside analog of deoxycytidine, gemcitabine (Galmarini et al., 2002), or the folate derivative methotrexate (MTX), which inhibits the enzyme dihydrofolate reductase (Chan and Cronstein, 2013) demonstrated great potency on cancer cells. Notably, MTX was specifically delivered to tumor cells by exploiting the hY_1_R-preferring peptide [F^7^,P^34^]-NPY. The modified conjugates [K^4^(GFLG-MTX),F^7^,P^34^]-NPY displayed high extracellular stability, paired with a fast uptake into cancer cells and the rapid release of MTX led to high cytotoxicity values in hY_1_R expressing tumor cells (Böhme and Beck-Sickinger, 2015). This potent cytotoxic effect was even further increased in MDA-MB-468 breast cancer cells by generating a double modified [K^4^(GFLG-MTX),F^7^,K^18^(GFLG-MTX),P^34^]-NPY conjugate (Böhme et al., 2016).

The third group of chemotherapeutics is formed by anti-mitotic agents, which act on microtubules. Drugs like paclitaxel (PTX), which inhibit microtubule depolymerization (Xiao et al., 2006), and vinca alkaloid analogs, which inhibit tubulin polymerization (Zhou and Rahmani, 1992), belong to this group. Even though PTX is highly hydrophobic and often associated with multidrug resistance owing to the P-glycoprotein-mediated efflux (Vargas et al., 2014; Barbuti and Chen, 2015), a PTX containing PDC reached phase 3 in clinical trials. The blood-brain barrier (BBB)-penetrating peptide angiopep-2 was loaded with three PTX molecules by ester linkers (ANG1005) (Régina et al., 2008). This highly loaded conjugate demonstrated higher brain uptake than free PTX and significant antitumor activity *in vivo* in glioblastoma-bearing mice (Régina et al., 2008). This conjugate also reached therapeutic concentrations in the tumor site and was well-tolerated in humans (Drappatz et al., 2013). Although no results of phase 2 were published yet, a phase 3 study was started in December 2018 (ANGLeD, 2018). Another very promising group of molecules is formed by kinesin spindle protein (KSP/Eg5/KIF11) inhibitors (KSPi), which affect the spindle formation during mitosis. When this process is impaired, cell cycle arrest is induced, which leads subsequently to apoptosis. Since these KSPis were found to be highly potent in various cancer types (Knight and Parrish, 2008; El-Nassan, 2013) their specificity has to be improved to transfer them into a clinical setting (Wakui et al., 2014). So far, only KSPi conjugated to antibodies were generated, which demonstrated high potency *in vitro* and *in vivo* (Lerchen et al., 2019).

### Radionuclides

Among chemotherapeutic drugs, radionuclides are the second major group of payloads in PDCs. They can be used for two main purposes related to cancer. The first field is their application in cancer diagnosis. Therefore, PDCs are labeled either with positron-emitting radioisotopes such as fluorine-18 (^18^F), copper-64 (^64^Cu), and gallium-68 (^68^Ga) to generate PET imaging agents or gamma-emitting radioisotopes including technetium-99m (^99m^Tc) and iodine-123 (^123^I), which can be used in single-photon emission computed tomography (SPECT). By binding to the targeted receptors on tumor cells, the malignant tissue can be precisely localized. The incorporation into the peptides is mostly facilitated by bifunctional chelating agents (BFCA), which provide the chelating group for the incorporation of the radiometals and a functional group allowing the conjugation to the peptide (Liu, 2008; Jamous et al., 2013). The most frequently applied BFCAs are diethylenetriaminepentaacetic acid (DTPA) and 1,4,7,10-tetraazacyclododecane-1,4,7,10-tetraacetic acid (DOTA) enabling the generation of PCDs with various radiometals and –lanthanides (León-Rodríguezn et al., 2008; Liu, 2008). Many reviews focused on the use of PDCs as molecular imaging agents and a huge variety of PDCs including a ^68^Ga-labeled bombesin analog (RM2), [^18^F]Galacto-RGD, ^99m^Tc-labeled [Phe^7^,Pro^34^]NPY and many more were investigated in a clinical setting (Schottelius and Wester, 2009; Sun et al., 2017).

Besides the diagnostic approach, radionuclide-labeled PDCs can also be seen in a therapeutic setting, if β-emitting, Auger electron-emitting or α-emitting nuclides are used (Karagiannis, 2007; Bhattacharyya and Dixit, 2011). The peptide receptor radionuclide therapy (PRRT) allows a directed and tissue-specific irradiation of tumor cells based on their overexpressed receptors. Many different radioactive metals are naturally or synthetically available and each of them features different properties. Therefore, the choice of the radionuclide strongly depends on the size of the targeted tumor or metastases (O'Donoghue et al., 1995).

The most commonly used radionuclides are indium-111 (^111^In), yttrium-90 (^90^Y), and lutetium-177 (^177^Lu) (Thundimadathil, 2012). While ^90^Y emits β-radiation with tissue penetration depths of up to 11 mm, ^177^Lu exhibits medium energy β-particles, which allow a tissue penetration range of max. 3 mm. Therefore, the use of ^90^Y is preferred for pronounced tumors due to the potential “cross-fire” effects, which can compensate for frequently occurring receptor heterogeneity (Goffredo et al., 2011). Nevertheless, ^177^Lu was found to eradicate small metastases better than ^90^Y because ^177^Lu emits besides β-particles also low-energy Auger electrons and conversion electrons (CEs) that deposit their dose over a short distance (Michel et al., 2005; Hindié et al., 2016). Another advantage of ^177^Lu is its low abundant gamma radiation, which enables post-therapeutic dosimetry.

These features are also provided by ^111^In, which is part of the first FDA approved radiopharmaceutical peptide-drug conjugate ^111^In-DTPA-octreotide (Octreoscan®) (Figure 7) (Kwekkeboom et al., 2010). Even though the use of high doses of Octreoscan® in patients with metastasized neuroendocrine tumors led to symptom relief, the tumor size regression was unsatisfactory (Ochakovskaya et al., 2001; Anthony et al., 2002). Therefore, Octreoscan® is only approved for diagnostic imaging of SSTR-positive tumors. To increase the biological effect of Auger electron-emitting isotopes, a delivery close to the nucleus has to be ensured. However, the most successful therapeutic peptide radiopharmaceutical so far is ^177^Lu-DOTA-TATE. This conjugate has been investigated intensively as PRRT-agent in several clinical centers in Europe (Hirmas et al., 2018). Treatment with ^177^Lu-DOTA-TATE resulted in longer progression-free survival and a significantly higher response rate than high-dose octreotide long-acting repeatable among patients with advanced midgut neuroendocrine tumors (Strosberg et al., 2017). The European Commission approved ^177^Lu-DOTA-TATE (Lutathera®) in October 2017, followed by the FDA in January 2018. This development demonstrates the enormous potential of the receptor-mediated drug delivery approach in cancer and is considered as a major milestone on the way toward peptide-based and personalized medicine.

## Boron Neutron Capture Therapy

Over the past decades, different approaches of targeted cancer therapy have been developed and reached the clinics. Even though these therapy approaches demonstrated excellent anti-tumor efficacy, the expected reduction in severe side effects was only partially achieved. Especially antibody-drug conjugates that target specific cell surface receptors display side effects including fever, nausea, infection, vomiting, stomatitis, and skin rashes (Hansel et al., 2010; Donaghy, 2016). These undesired unspecific effects are frequently mediated by the carrier molecules, which deliver their payload not only to the addressed tumor site but also to healthy tissues, which express the targeted markers as well. One possibility to overcome this issue is the use of non-toxic payloads, which have to be converted into their active form within the tumor cells. If the second step is also tumor-selective, the toxic payload will be generated only in tumor cells, whereas all healthy cells are not harmed because they contain only the inactive payload. This principle is used in a binary radiotherapy approach called boron neutron capture therapy (BNCT).

### Principles and General Requirements of BNCT

**Figure 8:**
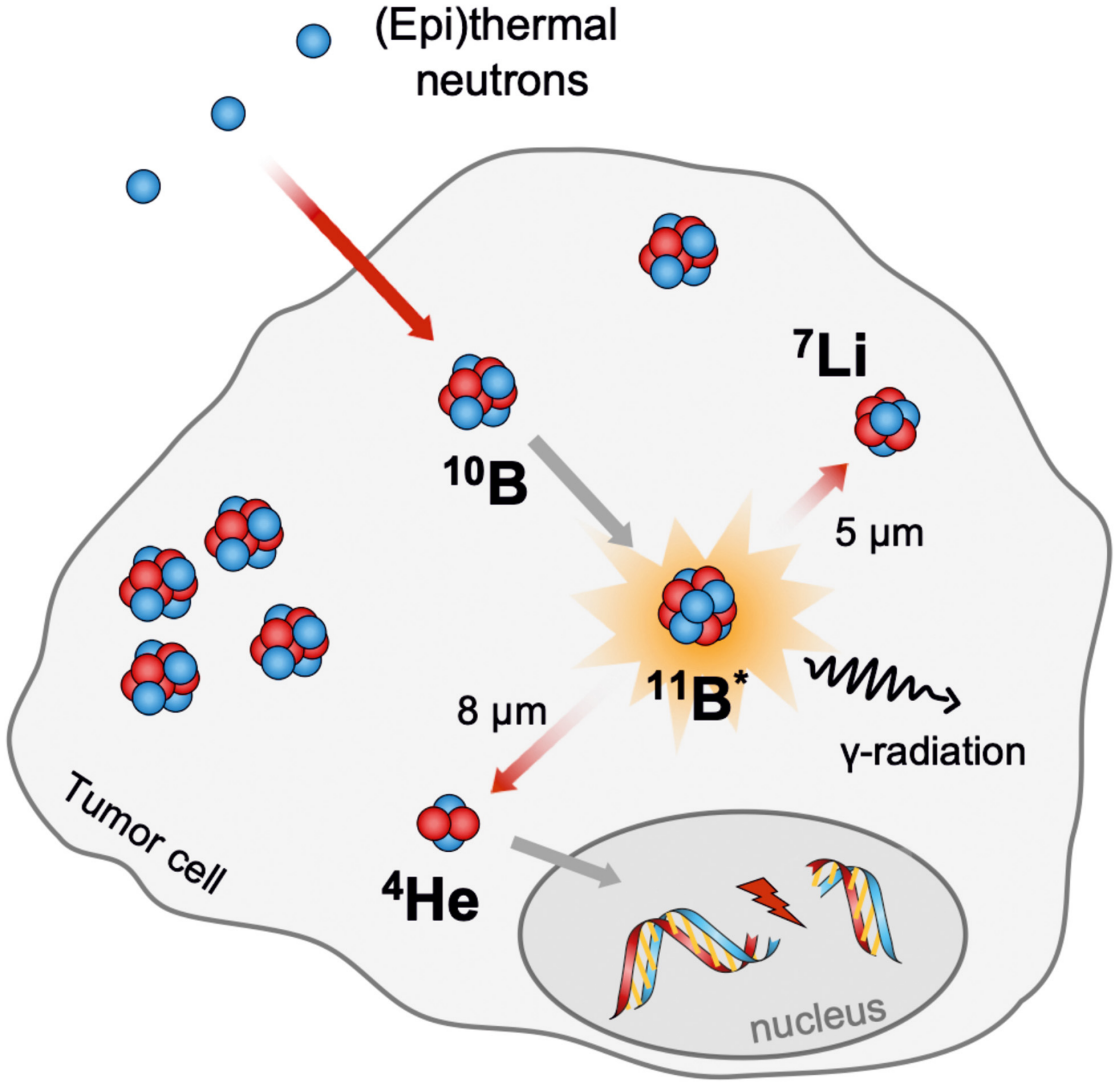
Schematic representation of the neutron capture reaction on boron neutron capture therapy (BNCT).

The idea of BNCT was already described by Locher (1936). In principle, BNCT offers the possibility to combine molecular drug targeting with the regional beam positioning of radiation therapy to achieve a double-selective therapeutic effect. This binary approach requires the accumulation of the non-radioactive isotope ^10^B, which comprises 19.9% of the naturally occurring boron in tumor cells. The subsequent local irradiation with thermal neutrons or epithermal neutrons (0.025 eV) induces the nuclear fission reaction [^10^B(1n,α)^7^Li] resulting in an excited [^11^B]^*^ nuclei, which decays immediately in high linear energy transfer (LET) particles (Locher, 1936). Due to the limited path lengths of the generated α and ^7^Li nuclei in tissues (5–8 μm), the destructive effects of those are limited to boron-containing cells (Figure 8) (Barth et al., 2005).

In order to achieve sufficient biological effects, the boron delivery agents must fulfill several critical requirements. (a) The uptake by tumor tissue has to be highly specific. To ensure no harm for non-neoplastic tissues, tumor to normal tissue and tumor to blood ratios of 3:1 and 5:1 are required, respectively. (b) Even though ^10^B features a remarkably high neutron capture cross-section of 3,840 barns, which is more than 3,000-fold higher than the neutron capture cross-section of ^14^N (1.82 barn) and ^1^H (0.332 barn) (Schmitt et al., 1960), a boron amount of 20 μg/g tumor is needed to achieve sufficient biological effects. This amount corresponds to about 10^9^ ^10^B atoms per cell, which could be lower if the boron is concentrated near or in the nucleus (Hawthorne, 1993; Hartman and Carlsson, 1994; Soloway et al., 1998). (c) The boron delivery agents have to retain within the tumor tissue over the period of neutron irradiation. (d) Simultaneously, the drug has to be rapidly cleared from the blood and healthy tissue. (e) The ^10^B-loaded agents must be chemically and metabolically stable and (f) must not feature any systemic cytotoxicity. (g) Moreover, they should have an appropriate water solubility.

Each of these demanding perquisites and especially their combination challenge the development of suitable boron delivery agents, limiting the establishment of BNCT as a viable cancer treatment modality in the clinic so far (Kreiner et al., 2016).

### Past and Recently Developed Boron Delivery Agents

**Figure 9:**
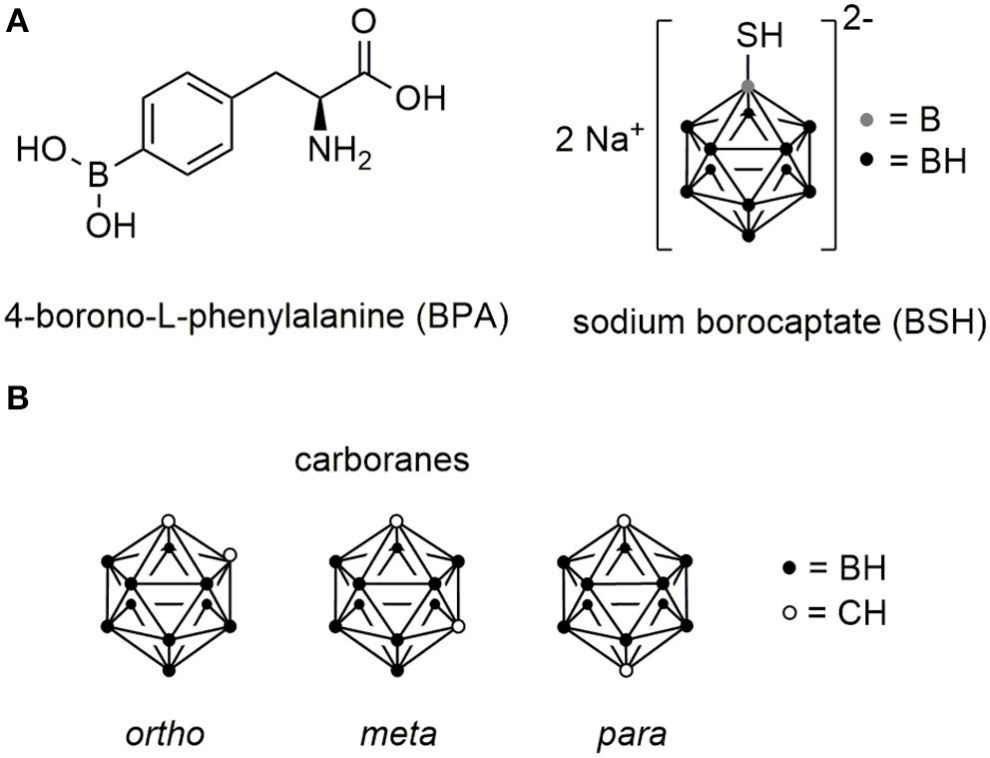
Chemical structures of boron cluster. **(A)** Structures of L-boronophenylalanine (BPA) and sodium borocaptate (BSH). **(B)** Chemical structures of *ortho-, meta-*, and *para-*carborane isomers.

After the first efforts, during the 1940s and 1950s, using the simplest boron salts including disodium tetraborate and sodium pentaborate, it became clear that a selective and high boron accumulation in tumor cells is crucial for an application in BNCT (Farr et al., 1958). Around the 1960s, l-boronophenylalanine (BPA) (Snyder et al., 1958) and sodium mercaptoundecahydro-*closo*-dodecaborate (BSH) (Soloway et al., 1967) were found to accumulate in the desired tissues in sufficient amount (Figure 9A).

BSH is a small hydrophilic molecule and has the advantage over BPA to deliver 11 boron atoms per molecule to the tumor. Although BSH has been applied for the treatment of glioblastomas demonstrating no toxic effects prior to irradiation, the efficacy has been limited due to low tumor boron concentrations (Kageji et al., 1997). To increase the boron concentration, various studies have been conducted in combination with other boron compounds (Barth et al., 2012).

Nowadays, BPA is the most applied drug in BNCT and is actively taken up into tumor cells by the L-type amino acid transporter system, which is highly expressed in tumor cells (Wittig et al., 2000). This results in higher boron concentrations within the tumor compared to BSH, which accumulates only passively in tumor tissue and cannot cross the cell membrane due to its net charge (Sköld et al., 2010). However, BPA has very limited water solubility (1.6 g/L) requiring the formulation as fructose complex, which demonstrated favorable biodistribution properties (Mori et al., 1989; Coderre et al., 1997). Even though BPA is considered to be a better boron delivery agent than BSH, it features some critical disadvantages. BPA consists of only a single boron atom per molecule requiring the administered of very high doses (500 mg/kg) and the rather modest tumor selectivity results in suboptimal BNCT efficiency (Luderer et al., 2015). Despite some promising results achieved by treating patients with recurrent head and neck cancer, high-grade gliomas and advanced melanomas, the development of novel boron delivery agents would lift this technology to the next level and support the implementation as advanced cancer treatment in clinics (Barth et al., 2018b). Although linear accelerator-based neutron beams have been developed, allowing the generation of neutrons in hospitals, boron delivery agents with high boron loading and excellent selectivity are still needed (Suzuki, 2019).

Multiple molecule classes have been investigated to find novel BNCT agents including low molecular weight compounds such as boronated nucleosides, amino acids, sugars, and porphyrin derivatives. Moreover, huge polymers like polyanionic and -cationic polymers and polyamines, as well as lysosomes and nanoparticles filled with boron were designed to facilitate extensive boron loading (Luderer et al., 2015; Barth et al., 2018a). To increase the selectivity of these polymers, biomolecules including peptides, proteins, and antibodies were attached to these structures allowing the directed targeting of cancer cells. EGFR has been studied extensively as a target in BNCT owing to its elevated expression in gliomas. Heavily boronated polyamidoamine dendrimers (around 1,000 boron atoms) were linked to the natural ligand EGF (Capala et al., 1996; Yang et al., 2009a) and the monoclonal antibodies Cetuximab (Wu et al., 2004, 2007) and L8A4 (Yang et al., 2006, 2008). *In vivo* studies of the latter two revealed that the combination of both antibodies is recommended to achieve a reasonable increase in mean survival times after irradiation in rats bearing tumors expressing both isoforms (EGFR and ERGFRvIII) of the EGFR (Yang et al., 2009b).

Besides EGF, peptides have also been investigated as potential boron delivery systems. In a number of studies, carboranes were used due to their favored properties. These icosahedral, hydrophobic C_2_B_10_H_12_ clusters have a high boron content, while they occupy a rather small amount of space, slightly larger than a rotating phenyl ring (Scholz and Hey-Hawkins, 2011). Three different isomers exist, which are defined by the position of the carbon atoms in the cluster (Figure 9B). Moreover, they can be chemically functionalized to readily allow the facile conjugation to peptides by peptide chemistry and they display high biological stability and relatively low cytotoxicity (Valliant et al., 2002).

**Figure 10:**
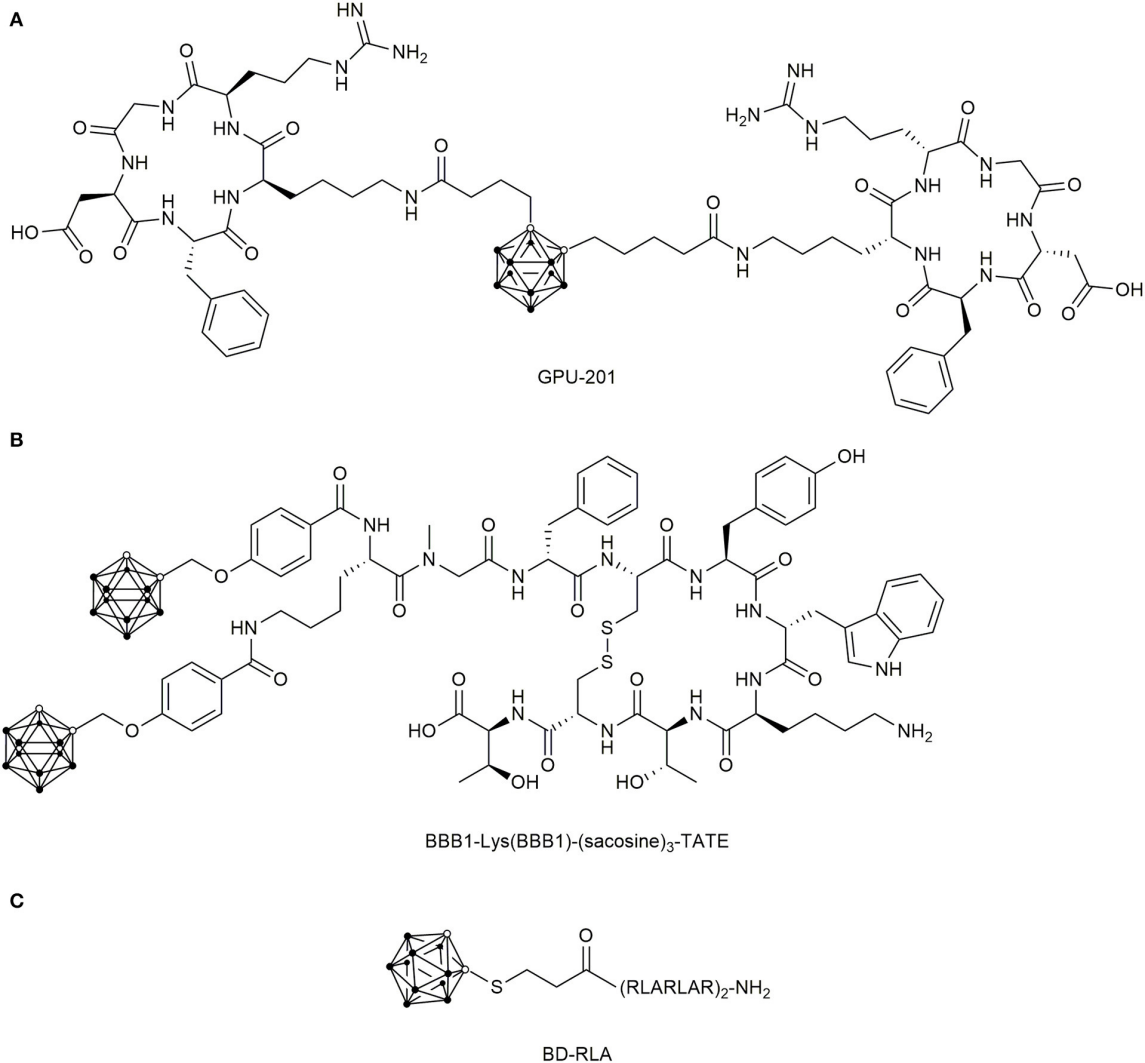
Chemical structure of boronated peptide-drug conjugates **(A)** Chemical structures of the dimeric carborane-c(RGDfK) conjugate GPU-201 for integrin αvβ3 targeting. **(B)** Double-carborane modified [Tyr^3^]-octreotate (TATE) for SSTR2 targeting. **(C)** Dodecarborate modified mitochondrial targeting CPP RLA.

In 2011, dimeric integrin targeting PDCs were designed (Kimura et al., 2011), which featured *ortho*-carboranes as center and two cyclic c(RGDfK) peptides that were separately conjugated via a butanoic acid linker to each carbon atom in the cluster (Figure 10A). The resulting conjugate, named GPU-201, displayed a high integrin αvβ3 binding affinity, dose-dependent tumor uptake in squamous cell carcinoma (SCCVII)-bearing mice and longer tumor retention time than BSH. Even though it showed a stronger tumor growth inhibition after irradiation compared to BSH alone, GPU-201 also demonstrated higher toxicity than BSH to both proliferating and quiescent cancer cells without irradiation (Masunaga et al., 2012).

The first boron delivery agent targeting cancer cells by their overexpressed GPCRs was proposed in 2003 (Schirrmacher et al., 2003). They used the SSTR2-addressing [Tyr^3^]-octreotate (TATE), which was N-terminally modified with the *ortho*-carborane derivative 5,6-dicarba-*closo*-dodecaboranyl hexynoic acid. Subsequently, they attached a BSH via Michael-addition to the N-terminus of TATE, both derivatives showed no biological activity (Mier et al., 2004). Four years later, Betzel et al. succeeded in creating the first biologically active and boronated TATE analogs by using the ortho-carborane-containing building block 4-(O-methylencarboranyl)-benzoic acid (BBB1), which was coupled either directly to the N-terminus or by glycinylsarcosine spacers of different length (Figure 10B) (Betzel et al., 2008). Even though double carborane modified TATE conjugates were generated with suitable SSTR2 affinity, further pharmaceutical development of SSTR2-targeting, boronated peptides were not undertaken.

**Figure 11:**
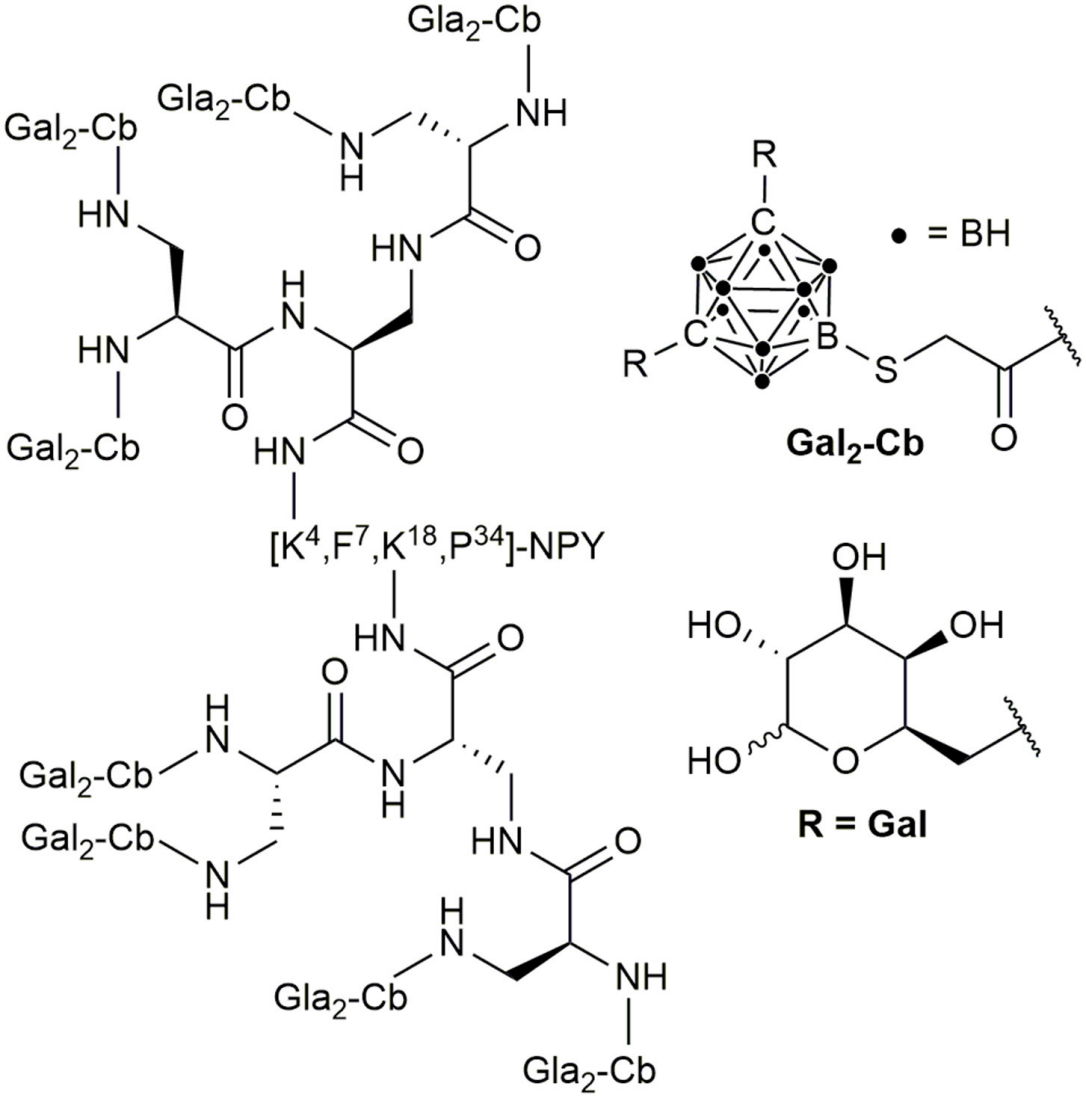
Chemical structure of a highly boron loaded [F^7^,P^34^]NPY conjugate.

In addition to these PDCs, the hY_1_R preferring peptide [F^7^,P^34^]-NPY has been recently described as a suitable target in BNCT. An *ortho*-carboranyl propionic acid (Cpa)-containing amino acid Fmoc-Lys-N_ε_(Cpa)-OH was incorporated at position 4 by solid-phase peptide synthesis (Ahrens et al., 2011). The modification led only to a slight loss of activity at the hY_1_R, but the hYR subtype-specific internalization was maintained. These results led to the development of the *ortho*-carborane building block 9-(carboxymethylthio)-1,2-dicarba-*closo*-dodecaborane(12) and a deoxygalactosyl-functionalized, charge-compensated cobalt bis(dicarbollide) building block, which were introduced at position 4, 18, or 22 of [F^7^,P^34^]NPY by the side-chain of substituted lysines at the respective positions (Ahrens et al., 2015; Frank et al., 2016). A triple modified conjugate with 30 boron atoms per peptide molecule demonstrated nanomolar potency at the hY_1_R and selectivity against other hYR subtypes and the sufficient uptake into hY_1_R transfected HEK293 cells was proven. Recent studies showed that introduction of sugar moieties to heavily carborane-loaded hY_1_R selective analogs can enhance solubility and thereby enable the boron conjugation of up to 80 boron atoms (Worm et al., 2019) (Figure 11).

The concept of boron-containing peptides was also investigated for ghrelin analogs and GRPR analogs. A ghrelin receptor superagonist was modified with different carborane monoclusters and the meta-carborane building block with a mercaptoacetic acid linker demonstrated the best results (Worm et al., 2018). The GRPR-selective ligand [d-Phe^6^, β-Ala^11^, Ala^13^, Nle^14^]Bn(6–14) contained multiple bis-deoxygalactosyl-carborane building blocks, which allowed the generation of a peptide conjugate with up to 80 boron atoms per molecule (Hoppenz et al., 2020). These modifications did not influence receptor activation but metabolic stability was increased and no intrinsic cytotoxicity was observed. Notably, undesired uptake into liver cells was suppressed by using l-deoxygalactosyl modified carborane building blocks.

Three studies reported the design and synthesis of CPPs modified with boron clusters. Conjugation of eight BSH clusters to the 11R peptide (8BSH-11R) facilitated the delivery of high amounts of boron (around 5,000 ppm/10^6^ cells) into U87ΔEGFR glioma cells (Michiue et al., 2014). In comparison to BSH, 8BSH-11R resulted in significantly stronger growth inhibition of U87ΔEGFR glioma cells after neutron irradiation. In addition, accumulation of 8BSH-11R in implanted cells in mice was observed, while no uptake in healthy brain tissue was demonstrated.

In another study, an arginine-tripeptide (3R) was modified with BSH and a DOTA chelator to allow ^64^Cu-radiolabeling and uptake quantification in glioma-bearing mice by PET imaging. For the labeled 3R conjugate, significantly higher tumor-to-normal-brain and tumor-to-blood radioisotope accumulation ratios compared to BSH-DOTA-64Cu were observed (Iguchi et al., 2015).

Moreover, the mitochondrial targeting peptides KLA and RLA peptides were conjugated to dodecaborates (DB) in a very recent study (Figure 10C) (Nakase et al., 2019). It was shown that DB-RLA reached higher boron concentrations in C6 glioblastoma cells than BSH and the cell mortality rate was significantly higher than for BSH after neutron irradiation, for which no cytotoxicity was observed. The number of the conducted studies and the variety of applied approaches demonstrate the pronounced interest in developing suitable boron delivery agents.

## Conclusion

Today, targeted delivery of anticancer agents remains one of the most appealing methods for cancer treatment. Peptide receptors are promising targets owing to their high overexpression in various tumor types. The synthetic analogs of natural peptide ligands are of major interest for PDCs as they possess high target affinity and specificity, fast internalization rates, and low immunogenicity. Previous limitations including low *in vivo* stability can be overcome by peptide modification and therapeutic cargos can be readily introduced to the peptides. PDCs have to compete with recently developed antibody-drug conjugates, but by now, they are rapidly catching up. More and more conjugates containing classical chemotherapeutic agents progress into clinical studies and in January 2018, ^177^Lu-DOTA-[Tyr^3^]-octreotate was approved by the FDA as the first PDC used in a therapeutic setting. This demonstrates the applicability of peptide-drug conjugates in future cancer treatment. Notably, the full potential of this approach is not exploited yet and PDC of the next generations are already in the pipeline. PDCs with intelligent linkers or non-radioactive payloads such as ^10^boron might not only provide even better tumor selectivity but also higher efficacy. Therefore, the approach of peptide-based and receptor-mediated drug delivery will give new impulses to cancer therapies in the future.

## Author Contributions

AB-S initiated the project. PH searched the data and wrote the manuscript. SE-H and AB-S revised the manuscript. All authors contributed to the article and approved the submitted version.

## Conflict of Interest

The authors declare that the research was conducted in the absence of any commercial or financial relationships that could be construed as a potential conflict of interest.
